# Supplementary material for: Cross-Checking Different Sources of Mobility Information
Source: PLoS One. 2014 Aug 18;9(8):e105184. doi: 10.1371/journal.pone.0105184 (PMC4136853; doi:10.1371/journal.pone.0105184)
Supplement: Supporting Information S1 — Supporting files and figures. (PDF) [file pone.0105184.s001.pdf]

Supporting Information: Cross-checking different sources of mobility information

Maxime Lenormand[1], Miguel Picornell[2], Oliva G. Cantú-Ros[2], Antònia Tugores[1], Thomas Louail[3,4], Ricardo Herranz[2], Marc Barthelemy[3,5], Enrique Frías-Martínez[6], José J. Ramasco[1]

1 Instituto de Física Interdisciplinar y Sistemas Complejos IFISC (CSIC-UIB), Palma de Mallorca, Spain

2 Nommon Solutions and Technologies, Madrid, Spain

3 Institut de Physique Théorique, CEA-CNRS (URA 2306), Gif-sur-Yvette, France

4 Géographie-Cités, CNRS-Paris 1-Paris 7 (UMR 8504), Paris, France

5 Centre d'Analyse et de Mathématique Sociales, EHESS-CNRS (UMR 8557), Paris, France

6 Telefónica Research, Madrid, Spain

Email address of corresponding author: maxime@ifisc.uib-csic.es

Mobile phone data pre-processing

1.1 Outliers detection

For both datasets we need to identify the outlier days to remove them from the data base. There are two types of outlier days, the special days (for example the National day) and the day for which we do not have the data for few hours. For example, for the metropolitan area of Barcelona, we can observe in Figure S1a eight days (from Monday to Monday) without outliers and in Figure S1b eight days with two outliers, Sunday, October 11th 2009 for which we do not have the data from 5PM to 11PM and Monday, October 12th 2009 the Spain's National Day.

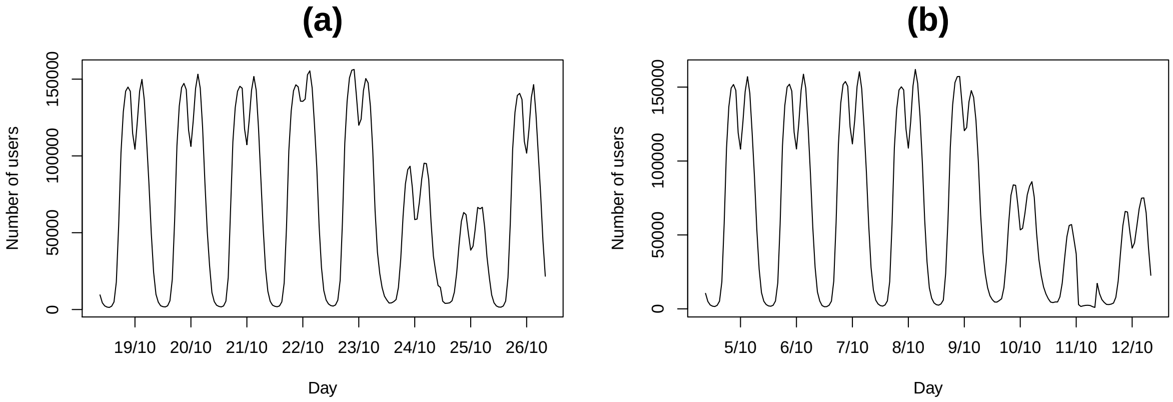

Figure S1. Temporal distribution of the mobile phone users for the metropolitan area of Barcelona. (a) From 19/10/2009 to 25/10/2009, eight days without outlier days. (b) From 05/10/2009 to 12/10/2009, eight days with two outlier days (11/10/2009 and 12/10/2009).

1.1.1 Voronoi cells

We remove the BTSs with zero mobile phone users and we compute the Voronoi cells associated with each BTSs of the metropolitan area (hereafter called MA). We remark in Figure S2a that there are four

types of Voronoi cells:

1. The Voronoi cells contained in the MA.
2. The Voronoi cells between the MA and the territory outside the metropolitan area.
3. The Voronoi cells between the MA and the sea (noted S).
4. The Voronoi cells between the MA, the territory outside the metropolitan area and the sea.

To compute the number of users associated with the intersections between the Voronoi cells and the MA we have to take into account these different types of Voronoi cells. Let m be the number of Voronoi cells, N_v the number of mobile phone users in the Voronoi cell v and A_v the area of the Voronoi cell v, $v \in |[1, m]|$. The number of users $N_{v \cap MA}$ in the intersection between v and MA is given by the following equation:

$$N_{v \cap MA} = N_v \left(\frac{A_{v \cap MA}}{A_v - A_{v \cap S}} \right) \tag{1}$$

We note in Equation S1 that we remove the intersection of the Voronoi area with the sea, indeed, we assume that the number of users calling from the sea are negligeable. Now we consider the number of mobile phone users N_v and the associated area A_v of the Voronoi cells intersecting the MA (Figure S2b).

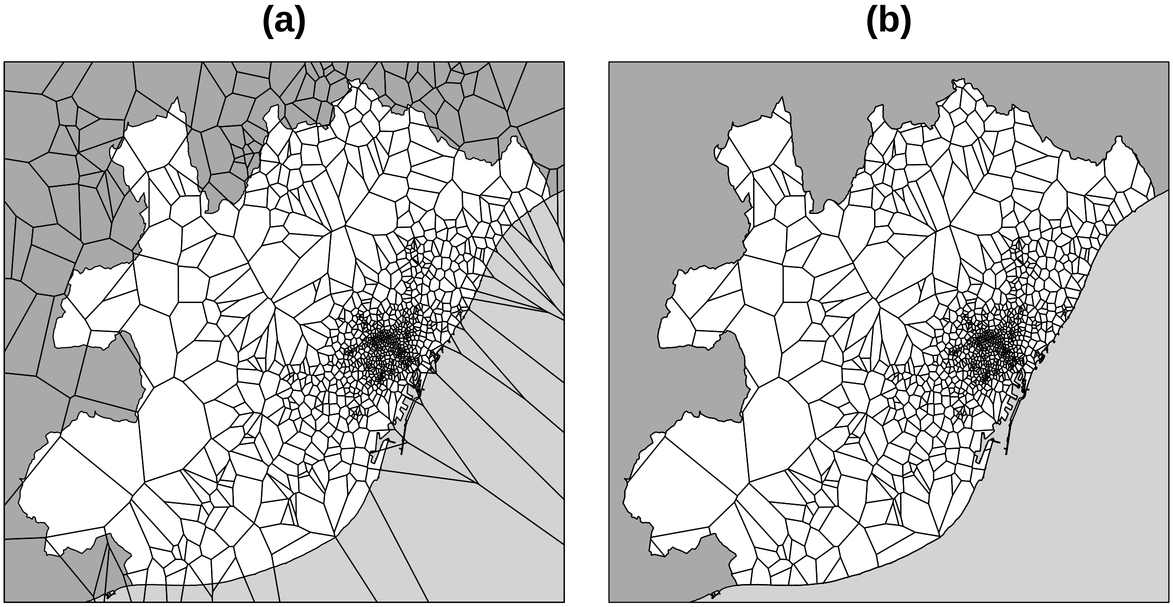

Figure S2. Map of the metropolitan area of Barcelona. The white area represents the metropolitan area, the dark grey area represents territory surrounding the metropolitan area and the gray area the sea. (a) Voronoi cells. (b) Intersection between the Voronoi cells and the metropolitan area.

Origin-Destination matrices

As mentioned in the section *Extraction of commuting matrices* unlike the Twitter data we cannot directly extract an OD matrix between the grid cells with the mobile phone data because each users' home and work locations are identified by the Voronoi cells. Thus, we need a transition matrix P to transform the BTS OD matrix B into a grid OD matrix G.

Let m be the number of Voronoi cells and n be the number of grid cells. Let B be the OD matrix between BTSs where B_{ij} is the number of commuters between the BTS i and the BTS j. To transform the matrix B into an OD matrix between grid cells G we define the transition matrix P where P_{ij} is the area of the intersection between the grid cell i and the BTS j. Then we normalize P by column in order to consider a proportion of the BTSs areas instead of an absolut value, thus we obtain a new matrix $\hat{P}$ (Equation S2).

$$\hat{P}_{ij} = \frac{P_{ij}}{\sum_{k=1}^{m} P_{kj}} \tag{2}$$

The OD matrix between the grid cells G is given by a matrices multiplication given in the following equation:

$$G = PBP^t \tag{3}$$

Supplementary figures

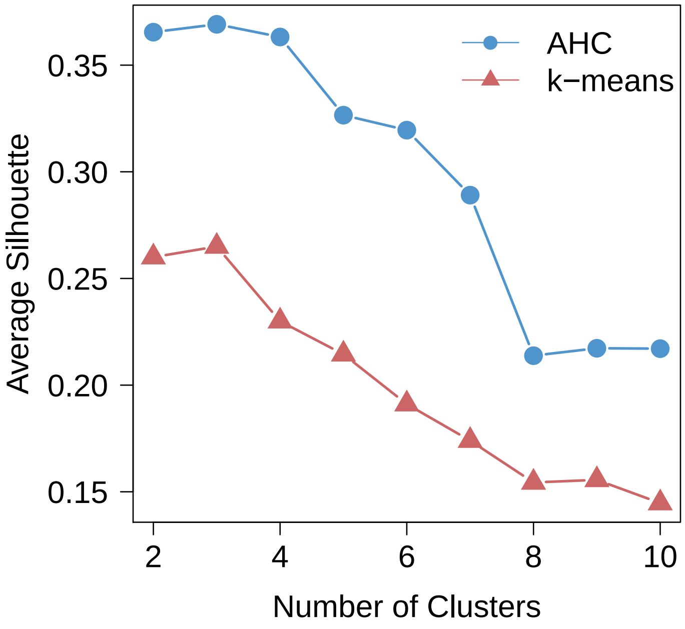

Figure S3. Average Silhouette as a function of the number of clusters obtained with AHC (in blue) and k-means (in red).

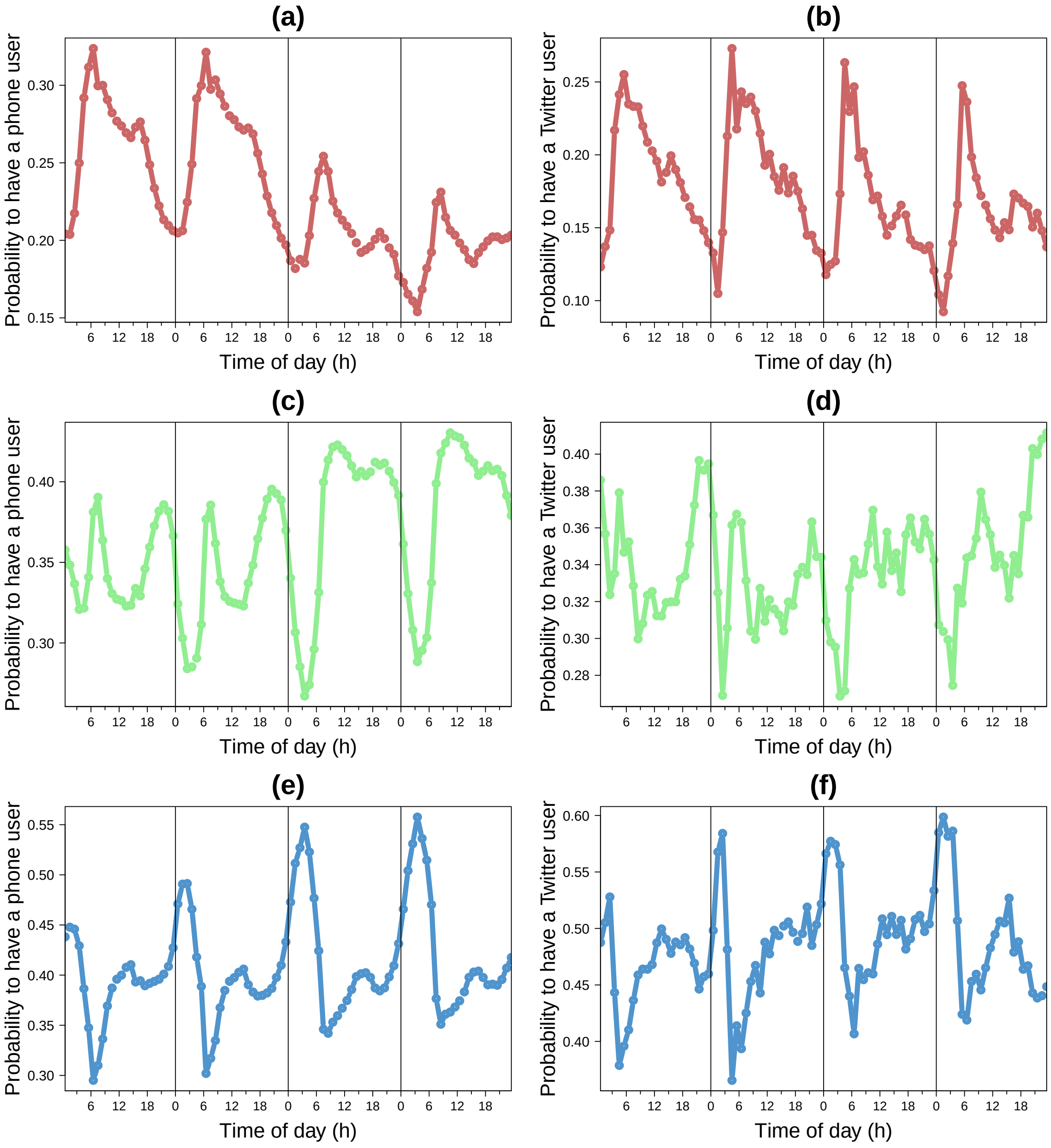

Figure S4. Temporal distribution patterns for the metropolitan area of Madrid ($l = 2$). (a), (c) and (e) Mobile phone activity; (b), (d) and (f) Twitter activity; (a) and (b) Business cluster; (c) and (d) Residential/leisure cluster; (e) and (f) Nightlife cluster.

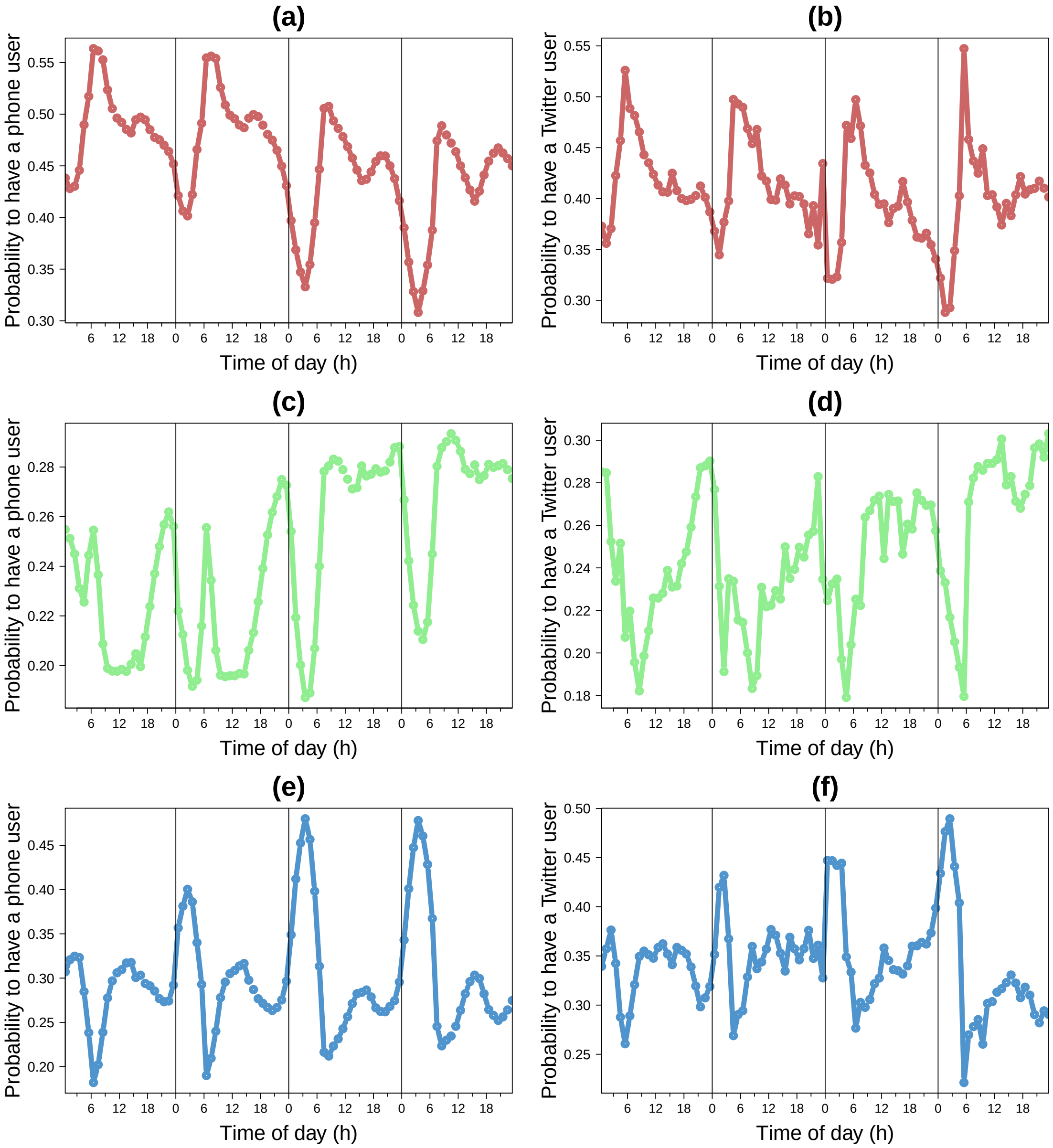

Figure S5. Temporal distribution patterns for the metropolitan area of Barcelona ($l = 1$). (a), (c) and (e) Mobile phone activity; (b), (d) and (f) Twitter activity; (a) and (b) Business cluster; (c) and (d) Residential/leisure cluster; (e) and (f) Nightlife cluster.

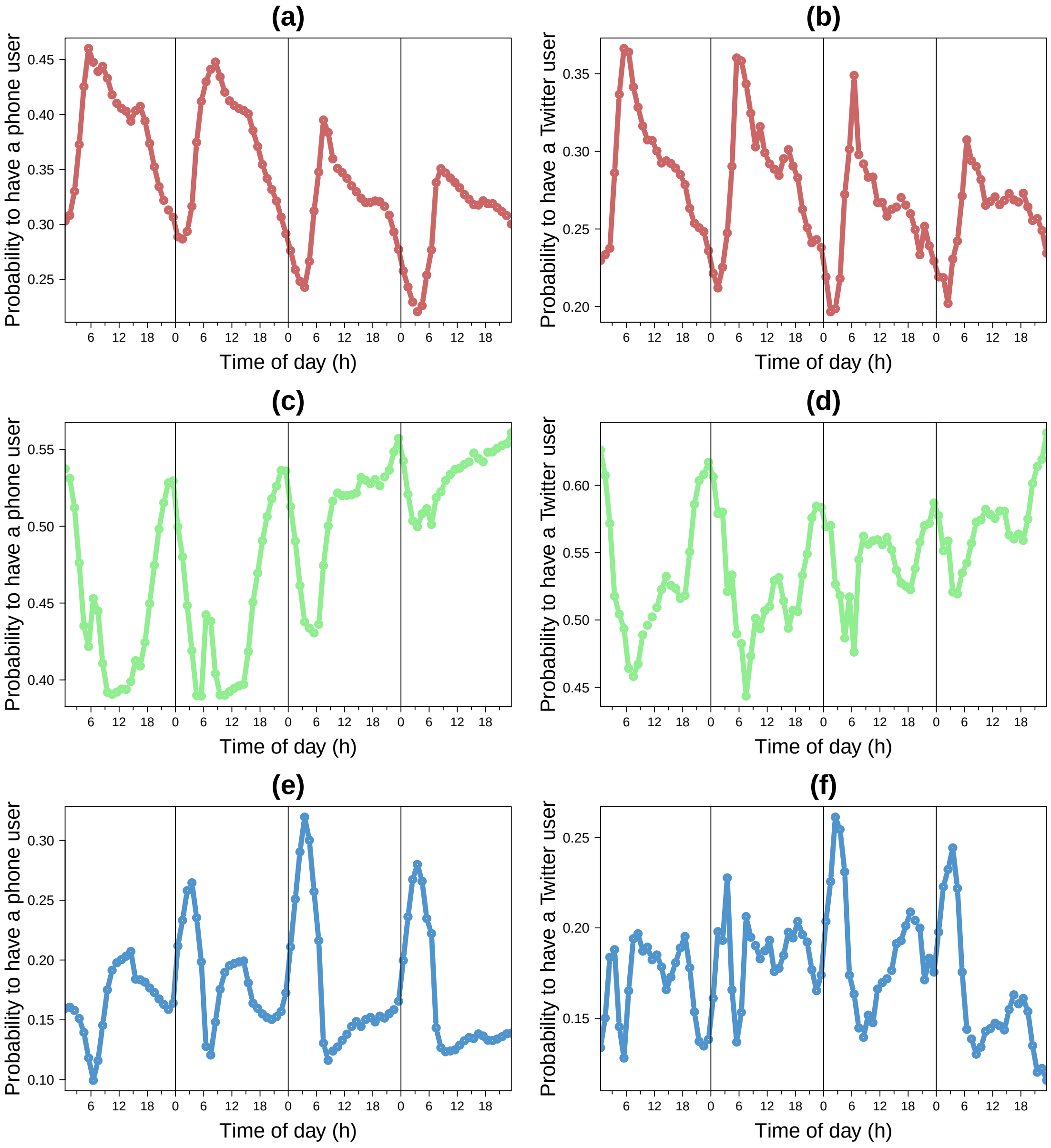

Figure S6. Temporal distribution patterns for the metropolitan area of Madrid ($l = 1$). (a), (c) and (e) Mobile phone activity; (b), (d) and (f) Twitter activity; (a) and (b) Business cluster; (c) and (d) Residential/leisure cluster; (e) and (f) Nightlife cluster.

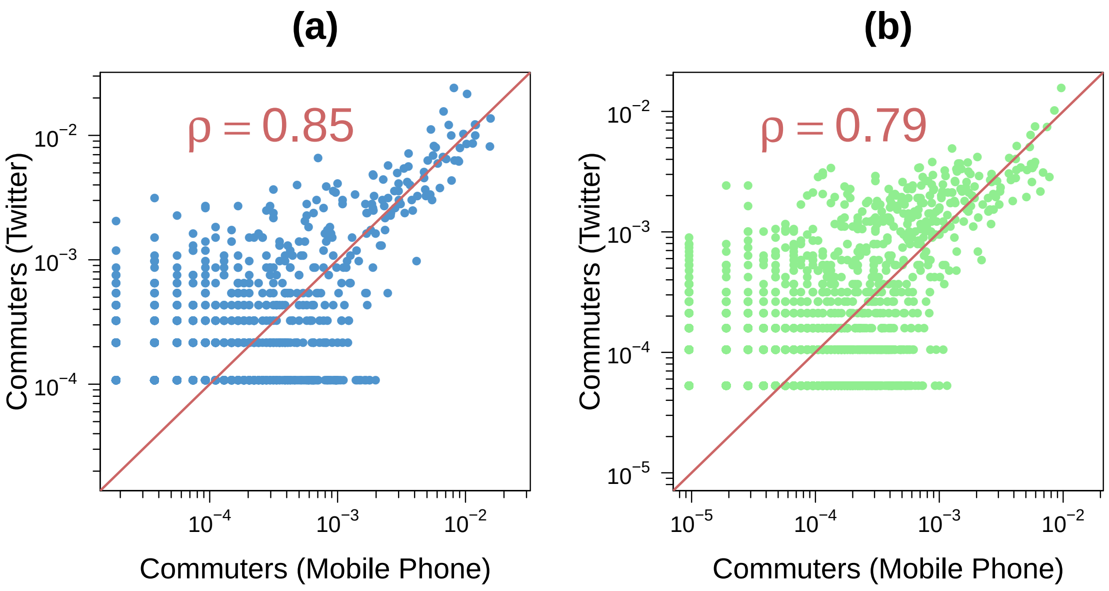

Figure S7. Comparison between the non-zero flows obtained with the Twitter dataset and the mobile phone dataset (the values have been normalized by the total number of commuters for both OD tables). The points are scatter plot for each pair of grid cell. The red line represents the $x = y$ line. (a) Barcelona. (b) Madrid. In both cases $l = 1$ km.

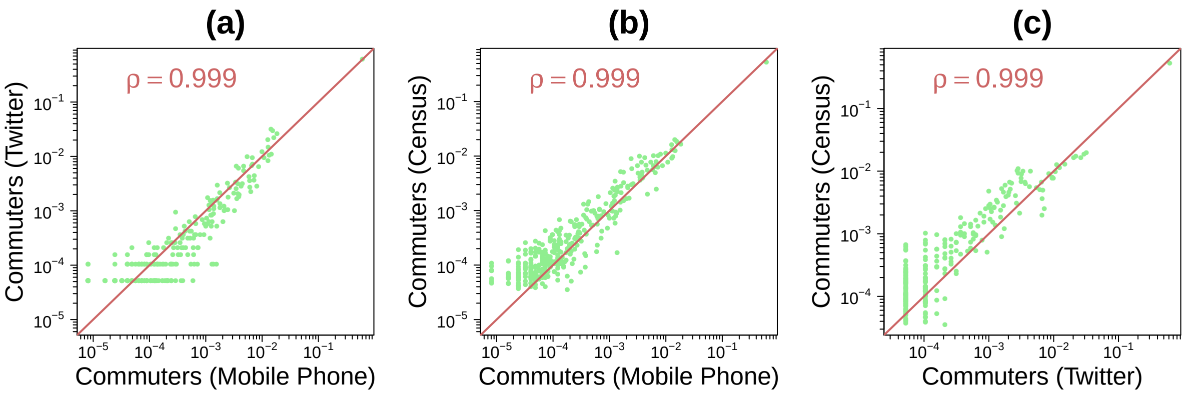

Figure S8. Comparison between the non-zero flows obtained with the three datasets for the Madrid's case study (the values have been normalized by the total number of commuters for both OD tables). Green points are scatter plot for each pair of municipalities. The red line represents the $x = y$ line. (a) Twitter and mobile phone. (b) Census and mobile phone. (c) Census and Twitter.
